# Neurofibromatosis Type 1-Associated Malignant Peripheral Nerve Sheath Tumor in the Nasal Cavity

**DOI:** 10.7759/cureus.45175

**Published:** 2023-09-13

**Authors:** Katerina Marini, James Philip Skliris, Konstantinos Garefis, Elissavet Skitotomidou, Nektarios Argyriou

**Affiliations:** 1 Department of Otorhinolaryngology-Head and Neck Surgery, 'G. Gennimatas' General Hospital, Thessaloniki, GRC; 2 Department of Pathology, 'G. Papanikolaou' General Hospital, Thessaloniki, GRC; 3 Second Academic Department of Ear, Nose and Throat (ENT), Papageorgiou General Hospital/Aristotle University of Thessaloniki, Thessaloniki, GRC

**Keywords:** otolaryngology, intracranial extension, nasal cavity, neurofibromatosis type 1, malignant peripheral nerve sheath tumor

## Abstract

Malignant peripheral nerve sheath tumors (MPNSTs) are rare soft tissue sarcomas, with 50% of cases associated with type 1 neurofibromatosis (NF-1). A 27-year-old male patient was referred to our department with an extended right nasal cavity MPNST. The lesion extended to the skull base, intracranial, parapharyngeal space, and infratemporal fossa. NF-1 was also confirmed by a neurologist. The patient was negative for distant metastases. Due to the tumor’s proximity to vital structures, it was decided to treat it with chemotherapy and radiotherapy. Nasal cavity MPNSTs are particularly uncommon, with few reported cases. They should be included in the differential diagnosis of nasal masses or recurrent nosebleeds, particularly in patients with NF-1. Careful follow-up is essential to detect early recurrence, which contributes to a better prognosis.

## Introduction

Malignant peripheral nerve sheath tumors (MPNSTs) are very rare soft tissue malignancies that arise from the peripheral nervous system. MPNSTs are characterized by nerve sheath differentiation and are commonly found in the trunk and limbs [1]. They have an incidence of 0.001% and account for 5-10% of sarcomas. However, only 15% are located in the head and neck (H&N) region [2]. Approximately half of the cases are associated with type 1 neurofibromatosis (NF-1). The optimal treatment involves complete surgical excision complemented by adjuvant radiotherapy, but the role of chemotherapy remains controversial. In this report, we present a relatively rare case of a right nasal cavity MPNST with extensions into the intracranial, parapharyngeal space, and infratemporal fossa in a 27-year-old male with a family history of NF-1. Due to the involvement of vital structures, the tumor was deemed inoperable. As a result, radiotherapy and chemotherapy were selected as the treatment modalities. Although the lesion initially decreased in size, at the six-month follow-up, it had further enlarged, exacerbating the patient’s symptoms. Consequently, palliative care was deemed the most appropriate course of action

## Case presentation

**Figure 1:**
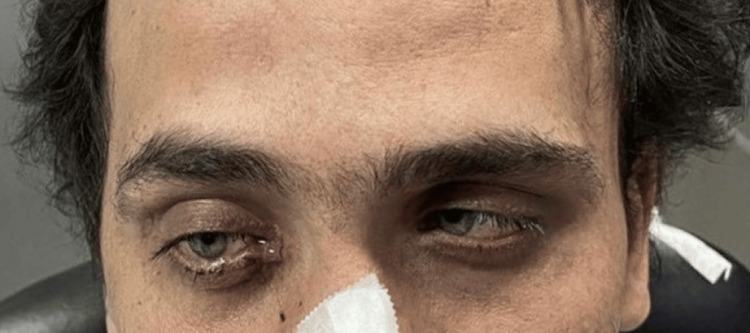
Image showing right exophthalmos and abducens nerve palsy in the patient.

A 27-year-old male patient was referred to our ENT department due to a history of nosebleeds that had persisted for four days. These were accompanied by diplopia lasting two weeks and impaired hearing in his right ear. His family history disclosed that his father had NF-1, and his brother suffered from an optic nerve glioma that led to blindness. The patient had previously been assessed by a neurologist at the age of three, owing to this significant family history, with no signs of NF-1, and he received no further medical intervention. At our department, a thorough physical examination was performed, revealing right exophthalmos and abducens nerve paralysis (Figure 1). 

**Figure 2:**
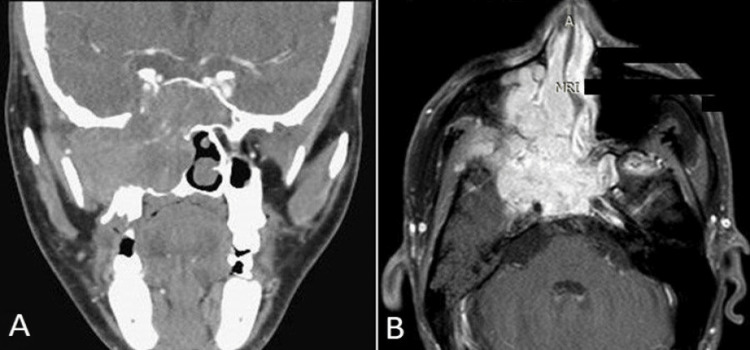
CT and MRI findings. (a) Coronal CT image showing a mass occupying the right nasal cavity with extensions into the parapharyngeal space, infratemporal fossa, and skull base. (b) Axial MRI image depicting the tumor encasing the right internal carotid artery.

Café-au-lait spots were observed on his neck, trunk, and arms, but neck palpation was unremarkable. Anterior rhinoscopy revealed an extensive, delicate mass in the right nasal cavity. Otomicroscopy showed right middle otitis with effusion. CT scan and MRI of the head and neck with contrast were performed, revealing a contrast-enhanced, heterogeneous, and extensive lesion. This lesion involved the right nasal cavity, right choana, and right paranasal sinuses and extended into the intracranial, infratemporal fossa, parapharyngeal space, skull base, and right cavernous sinus (Figures 2a-2b). It also encased the right internal carotid artery.

**Figure 3:**
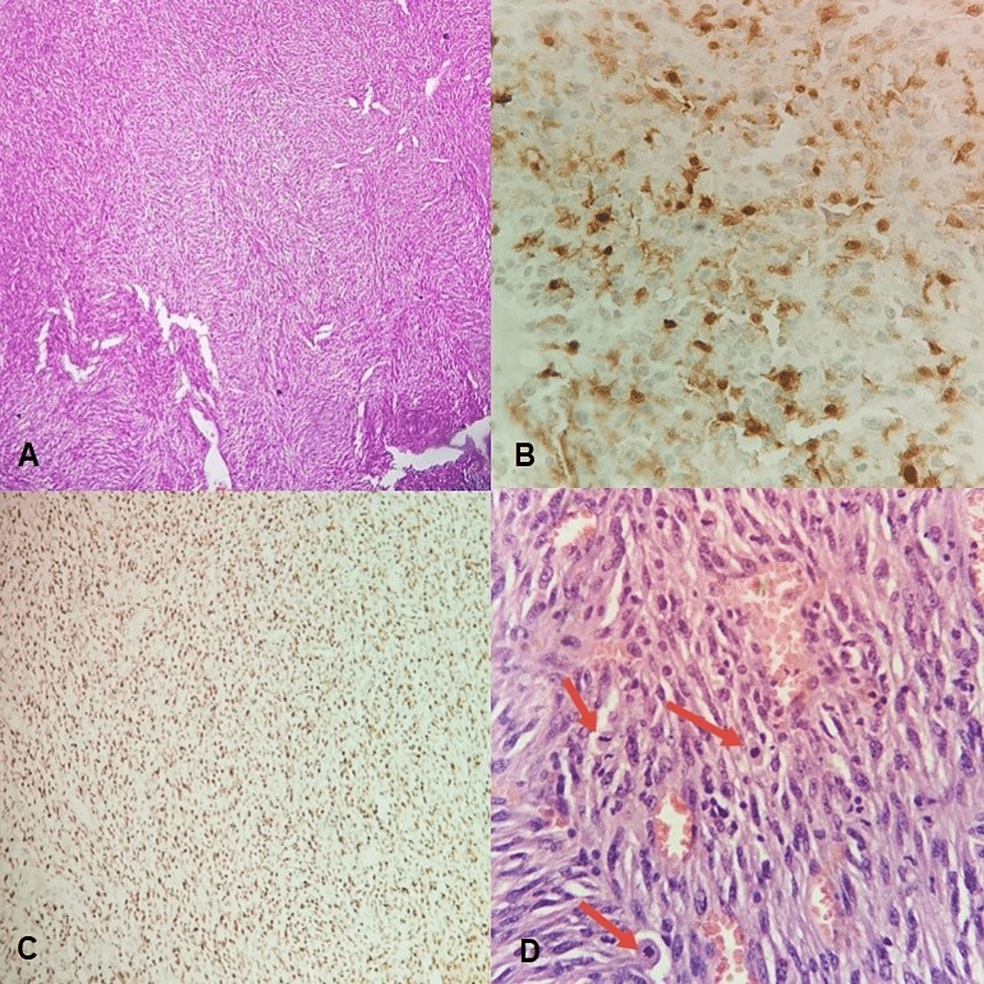
Histopathological and immunohistochemical examinations. (a) Evident hypercellularity at low power. A herringbone pattern of organization, not typical for this tumor, is visible among cellular whorls and fascicles (H&E, 4X). (b) Focal positivity of neoplastic cells with S-100 stain (IHC, 40X). (c) Proliferation index Ki-67 is very high, around 90% (IHC, 10X). (d) At high power, mostly mild atypia and frequent mitotic figures (arrows) are observed amidst vascular spaces (H&E, 40X). IHC: Immunohistochemistry.

A biopsy was performed under local anesthesia, followed by nose packing. However, a few days later, the mass had further enlarged, leading to a noticeable deviation of the soft palate through the oropharynx. The patient began experiencing worsening headaches and was subsequently referred to a pain clinic. He also reported dysphagia and weight loss. Following a neurologic assessment, he was diagnosed with NF-1. Histopathological and immunohistochemical examinations confirmed the presence of an MPNST (Figures 3a-3d).

CT scans of the brain, chest, and abdomen revealed no distant metastases. Due to the lesion's intracranial extension and involvement of vital structures, it was deemed inoperable. Therefore, a combination of chemotherapy and radiotherapy was chosen. Initially, the patient completed five cycles of chemotherapy with epirubicin and ifosfamide. He was also scheduled to receive 34 sessions of 68gy radiotherapy in conjunction with chemotherapy using cisplatin. While he exhibited clinical improvement during the first three months, a six-month follow-up revealed an increase in the size of the mass. Consequently, tumor recurrence was detected, leading to the cessation of both chemotherapy and radiotherapy. Given the unfavorable prognosis, the decision was made to transition to palliative care.

## Discussion

H&N sarcomas represent 2-15% of all sarcomas and 1% of H&N adult malignancies [1]. MPNSTs are an uncommon subtype, comprising 5-10% of sarcomas [2]. They arise from the peripheral nervous system, exhibiting nerve sheath differentiation (Schwann cell, fibroblasts, or perineural cell). The most common locations are nerve roots and bundles in the extremities, trunk, and pelvis [2]. MPNSTs located in the H&N region constitute 12-19% of all cases and are even rarer in the nasal cavity. Approximately half of the patients with MPNSTs have an association with NF-1 [3]. NF-1, also known as von Recklinghausen's disease, is an autosomal dominant disorder predisposing individuals to various benign and malignant tumors. Distinctive characteristics of these patients include café-au-lait macules, Lisch nodules, intertriginous freckling, and either dermal or plexiform neurofibromas [4]. Malignant transformation is primarily associated with deep neurofibromas [5]. MPNSTs have a prevalence of 10% among NF patients [6]. Additionally, about 10% of patients have a history of radiation exposure, while the remaining cases are sporadic [2]. Sporadic cases typically manifest between the ages of 20 and 50, whereas NF-1-associated MPNSTs tend to present about a decade earlier, with no gender predilection [7]. They can arise in various H&N locations, including the neck soft tissue, parotid, submandibular and minor salivary glands, preauricular area, mandible, lips, nasal cavity, paranasal sinuses, larynx, orbit, cranial nerves, and thyroid gland [3,8]. Regarding their clinical presentation, MPNSTs are characterized by paresthesia, motor weakness, pain, or the presence of enlarging masses [8]. When involving the nasal cavity, naso- and oropharynx, they may manifest as unilateral nasal obstruction, recurrent epistaxis, hyposmia, swelling, atypical pain, headache, rhinorrhea, exophthalmos, or nerve impairment [7].

MPNSTs lack a specific immunohistochemical profile, and markers such as S100 protein and SOX10 have low sensitivity and specificity, making the diagnosis challenging. Most tumors display high mitotic activity, intersecting fascicles, and monomorphic spindle cells, while foci of rhabdomyoblastic or cartilaginous differentiation may be present [3]. Differential diagnosis includes soft tissue tumors such as fibrosarcoma, synovial sarcoma, leiomyosarcoma, and melanoma [8]. When evaluating nasal masses, the differential diagnosis includes lymphoma, melanoma, nasopharyngeal carcinoma, juvenile angiofibroma, polyps, dermoid cyst, glioma, and encephalocele [9]. MPNSTs have the highest local recurrence rates among sarcomas, ranging from 40-65%. They have a propensity for distant metastases (approximately 40%) and exhibit rare lymph node involvement [6,8,10]. Often, they are diagnosed at an advanced stage, having already spread to other organs, primarily the lungs and bones [6]. The treatment of choice for MPNSTs is complete surgical excision with clear margins, either with or without adjuvant radiotherapy. However, the role of chemotherapy remains controversial [3]. H&N MPNSTs often involve critical structures and extensive surgical excision can result in significant defects. H&N location, early recurrence, large tumor size, metastasis, and the NF-1 mutation are all indicators of a poor prognosis [4,10]. The five-year survival rates range between 13-63%, but the overall prognosis, as well as the rate of local recurrence for MPNSTs, are generally considered poor [10].

## Conclusions

Otolaryngologists should always include sarcomas in the differential diagnosis of nasal obstruction, recurrent nosebleeds, rhinorrhea, worsening headache, or nerve impairment. Patients with NF-1 and the aforementioned symptoms should be thoroughly examined for the exclusion of MPNST, as early diagnosis and intervention lead to a better prognosis. Imaging is paramount for the extension of the lesion and distant metastases. Although the treatment of choice is complete surgical excision, involvement of vital structures at the H&N region is not rare, so radiotherapy with or without chemotherapy should be considered.
